# Exploring Gamification and Serious Games for Mental Health: A Narrative Review

**DOI:** 10.7759/cureus.74637

**Published:** 2024-11-27

**Authors:** Anantha Eashwar V M, Shirley Esther Pricilla, Aishwarya P M, Krishna Prasanth

**Affiliations:** 1 Community Medicine, Sree Balaji Medical College and Hospital, Chennai, IND

**Keywords:** depression, gamification technique, mental health apps, mental health problems, video games

## Abstract

The development of serious video games using gamification techniques and applying them to the management of mental health problems has emerged as one of the significant innovations in technology and mental health. However, various issues exist from the design stage of video games to the implementation stage, which can lead to problems with usability and accessibility and have non-beneficial effects on the individual. This review article provides an overview of various gamification technologies currently used in video games and virtual reality-based video games. It discusses their strengths and limitations in managing common mental health problems like depression, anxiety, and stress, as well as their application in non-communicable diseases such as cancer, diabetes, and obesity, along with improving psychosocial well-being. This article highlights the technology currently available and the need to address the lacunae in effectively utilizing technological advancement in the development of serious video games using gamification strategies to manage mental health problems.

## Introduction and background

Mental health problems and disorders are the major disorders worldwide and the leading contributors to mortality, morbidity, and years lived with disability [1]. Among these mental health problems, depression ranks as one of the major contributors to disability globally [2]. The economic costs required to treat mental health conditions far exceed the expenses necessary to treat other non-communicable diseases (NCDs) like cardiovascular diseases [3]. Due to rapid urbanization, psychological distress is more common among people due to rapid advancement in technology and lifestyle changes due to changes in the work atmosphere, sedentary lifestyle, and substance abuse like alcohol and tobacco dependence. In developing countries like India, people seldom consider turning to any psychiatrist or psychologist for common mental health problems like depression and anxiety, which in turn leads to underdiagnoses of those conditions. In developed countries, mental health-seeking behavior and acceptance are way better due to a lack of cultural stigma and acceptance of medication. If those problems are identified and treated, compliance is low due to physical and sociocultural barriers like acceptance of treatment, tolerance of side effects of drugs, and a widespread notion that mental health conditions do not warrant a visit to the doctor. In developed countries [4], computer video games are played by billions of people across the globe, with over 45% of the Indian population (major age group: 19-24 years) playing them in one way or another. Computer video games vary enormously based on types, genres, involved technologies, interactions, and goals. Quality computer games are shown to increase concentration, play a significant role in the retention of information, and facilitate deep learning. Due to the buzz in social media and the availability of entertainment in all forms and formats, the inclusion of computer games as an educational tool has gained attention in the present decade [5,6].

Computerized game-based approaches, categorized as “serious games” and termed “gamification,” have been used to motivate, educate, and persuade users in health, education, or other scenarios. Gamification refers to the application of the addition of game elements in non-game contexts. A gamified intervention may not operate as a whole game experience but contain gaming elements, such as scoring points, in-game rewards, or engaging in quests. Approaches that involve game-based elements are still in their infancy. Many studies done in various domains of mental health (like depression, anxiety, and stress) involving gamified aspects (leaderboards, scoring) have been proven to be effective in bringing about behavior and psychological changes, which proves that applied games with mental health components can impact behavior change in people suffering from mental health problems [7].

## Review

Serious games for depression

The available literature regarding the use of serious games in the treatment of mental health problems like depression is limited. Though studies have been done elsewhere, evidence regarding the use of the application of serious games in depression is lacking in countries like India.

Cognitive Behavioral Therapy (CBT) and Gamification: Smart, Positive, Active, Realistic, X-factor Thoughts (SPARX)

SPARX, a computerized cognitive behavioral intervention, was evaluated by Merry et al. using a randomized controlled non-inferiority trial among people suffering from depression. SPARX was an interactive fantasy game that was designed to provide CBT for the treatment of clinical depression. The game was an adventure game consisting of seven modules that were to be completed sequentially. The player chooses a random avatar and must undertake a series of challenges to restore balance to the fantasy world dominated by gloomy negative automatic thoughts (GNATS). Pre- and post-intervention depression scores were assessed. It was found that the mean score of depression was found to be significantly lower in the intervention arm compared to the treatment as a usual arm. It was more concentrated on the CBT aspect of treating depression. It was more of an action game than a simulation game [8].

Narrative-Driven Games: Obliti and Reach Out Central

Obliti was developed as a first-person game that follows the life of a person with depression. The game was developed based on the experiences of the author, who had post-traumatic stress disorder (PTSD) and depression. The game environments were depicted in grey color with frequent flashbacks that were in color, depicting how depression started in his life. The game uses Unreal Engine 4^TM^ as the development platform [9].

“Reach Out Central” was a serious narrative game developed and evaluated by Shandley et al. for improving the mental health of people aged 18 to 25 [10]. The primary purpose of the game was to provide narrative stories and questlines on dealing with the problems of alcohol use. The serious game was evaluated using a quasi-experimental design, proving that all the outcome measures improved post-implementation of the intervention. The major limitation included an open-ended trial design and a small number of male participants.

*Exploring* *Depression in Games: Depression Quest, Elude, and Others*

“Depression Quest” is a text-based game that simulates the life of a person living with depression. The player will be given a series of everyday tasks in the game, which they must clear along with managing the illness. The game is available free of cost. The major disadvantage of the game was that the game did not have any scientific background grounded in depression [11].

Applications of serious games beyond depression: cancer and virtual reality (VR)-based interventions

Other games that don’t have a scientific background but have concepts of depression in them include serious games like “Elude,” which was a symbolic representation of experiences associated with depression [8]. It begins in a dark forest, which is symbolic of the “default” mood of many people suffering from depression, which includes lethargic, cloudy, and uninterested in the world. As the player character walks forward (this is a 2D experience), he encounters birds (things he enjoys), and he can “resonate” with them to jump higher than he could otherwise. The goal, then, is to progress upward by jumping on tree branches until you get above the tree line. But he cannot climb up if he makes a mistake and falls. The metaphor here is that “a depressed person can pull himself out of the pits sometimes, but the slightest push will drop him down to the emotional depths with which all depressed people are familiar. And falling hard enough can leave you in a hole too deep to climb out of” [12,13]. “Actual Sunlight” is a top-down 2D game in which the player assumes the role of a person who has developed hatred toward his life. The game's storylines involve how the person goes on and does everyday tasks [14]. But the game's major disadvantage was that it was depressing to play, as the main storyline involved a paranoid person who blames everybody and does not take responsibility for anything happening in his life. The game's objective is to find out the deep-rooted problems that were the cause of his behavior. The significant issue in serious games like Actual Sunlight and Elude was that they were primarily made as video games with “depression” as a backdrop.

Serious games were used in breast cancer patients in treating moderate to severe depression using functional neuroimaging techniques. “Hit the Cancer” was a serious game given to the gaming group along with regular treatment, and the non-gaming group received only regular treatment. Post-intervention, the depression scores were found to be lower in the gaming group with a statistically significant association (p < 0.05) [15].

In a study done by Jouriles et al., validation of a virtual role play using VR models to assist females in resisting sexual attacks by males was done, which showed that, when compared with non-VR staged scenarios, VR models provided more involvement emotionally by females. He concluded that this could be used to provide training to women to develop behavioral strategies in resisting sexual advances by males using a VR model employing role-playing game design [16].

Gamification among schizophrenia patients

Schizophrenia is one among the many mental health conditions that affect the social life of an individual, affecting memory, attention, concentration, and various executive functions of the individual. A study by Shimizu et al. explored the effect of an interactive video game (IVG), Nintendo Wii™ Sports Resort, on the frontal lobe functions in schizophrenia patients. It was found that there was an improvement in the health-related quality of life among patients who played video games and an increase in the activation of the prefrontal cortex compared with the group that received conventional treatment. This shows that video games can be tried as a low-cost rehabilitation among schizophrenia patients [17].

Role of serious video games in alleviating anxiety

Anxiety disorders are one of the many significant mental health problems among children and youth, affecting up to 22% and 49% of the population, respectively [18,19]. Applied games have garnered attention as a cost-effective delivery model for preventing anxiety. A study done by Scholten et al. employed a randomized controlled trial (RCT) design in which “Dojo,” a video game that was specifically designed to reduce anxiety among adolescents, was used. It includes two crucial evidence-based anxiety-reducing mechanisms: heart rate variability (HRV) and emotion regulation training. The study found that there was an improvement in anxiety symptoms among the group that received the video game intervention [20].

Another video game specifically designed for children with anxiety was “Mindlight.” It is a survival horror game that helps children cope with anxiety. It is based on the principles of neurofeedback, attention bias modification, and exposure training. A study by Schoneveld et al. found that anxiety symptoms were reduced in children who played video games at three-month follow-ups [21]. Similarly, when Mindlight and CBT were delivered in separate arms of an RCT, the reduction in anxiety-related symptoms was the same in both arms [22].

Use of video games in impulsive disorders

A team of programmers, clinicians, and engineers developed a video game prototype called PlayMancer. In it, the player is introduced to a scenario where his final goal is to develop self-control skills to handle his impulsive behaviors. The biofeedback mechanism was used to help the players learn various skills related to self-control, relaxation, and impulsive behaviors by performing various in-game tasks like climbing, diving, and relaxation. The study found that this video game was effective for people suffering from eating disorders (ED) and impulsive behaviors and suggested that video games were one of the best therapies to complement traditional management [23].

VR, gamification, and mental health

Imaginal exposure therapy is a psychological therapy used to help in overcoming their fears. It is effective in managing people with PTSD, phobia, and obsessive-compulsive disorders (OCD). Using the technology of VR, various real-life scenarios can be effectively simulated in a computer-generated environment in which the player can interact as if in the real world. Combined with concepts of gamification, various studies reported that VR-based therapy was more influential in treating these disorders than imaginal exposure therapy. Different mental health conditions have benefitted from integrating VR for effective management and control. Anxiety-related conditions like public speaking anxiety and generalized anxiety disorder have been treated by providing controlled environments in VR exposure therapy and CBT. Patients also reported a high level of satisfaction with the inclusion of VR-based approaches in the treatment of PTSD, OCD, and phobias [24,25].

Depression among older people, especially late-onset depression, has a higher tendency to become chronic. It can irreversibly affect the quality of life of an individual if not treated at an early stage. Storytelling using VR, metaphorically reflecting the emotional state of the individual and showing them solutions, has been shown to reduce the intensity of depression-related symptoms coupled with the reduction in anxiety levels among older women [26].

Use of gamification in the management of lifestyle diseases

“Mission: Schweinehund” was a self-explanatory game that uses self-determination theory as the theoretical framework. The theory states that when the three basic human needs, namely, autonomy, competence, and relatedness, are met, the individuals become motivated and engaged in activities with full enthusiasm. The goal of the video game was to plant trees and flowers, helping the restoration of a garden, which in turn attracts animals living in the garden, which comes back, helping the restoration process. In this video game, the garden's restoration metaphorically represents the restoration of the player’s body through regular physical activity (PA). Various motivational elements and behavior change techniques were incorporated into the video game. An RCT was conducted by Höchsmann et al. among type 2 diabetes patients, with this video game as an intervention in one arm. Among type 2 diabetes patients who were physically inactive, incorporating this video game as a motivational and behavior change tool significantly increased PA motivation and adherence for 24 weeks [27].

“Exergames,” or active video games (AVG), require the person to physically move and perform some actions to interact with the images on the screen through bodily movements to complete the level in the video games. As the player progresses, more difficulty levels will be unlocked, making the player burn more calories by performing more physical movements based on the level design. These games have proven to improve cardiovascular health and physical fitness, coupled with the betterment of their psychological well-being, and help to act as a potential tool for the treatment of obesity [28].

Enhancing patient engagement through gamified mental health interventions: advantages and disadvantages

In the management of mental health disorders, patient engagement and loss of follow-up due to lack of motivation among patients are some of the major concerns in the effective treatment of mental health disorders. Some of the causes include adverse effects of drugs, feeling the doctor is too judgmental, and stigma in the family and social circle. This will lead to the ineffectiveness of the treatment, further worsening both the condition and the attitude of the patient toward mental health-seeking behavior [29].

The significant advantage of using gamified mental health intervention is that it could address this problem and decrease attrition in the management of mental health disorders by using gaming mechanics to motivate the players, using badges and medals, and making the whole process more engaging, enjoyable, and rewarding. The disadvantage of using video games is the significant cost incurred to develop the video game and the training to be provided to the patients to effectively engage with them [30].

Though patient engagement may be beneficial in most cases, over-engagement in gamified interventions can lead to a sense of addiction to earning badges and completing levels in the video game, which may lead to neglecting other important activities in daily life, and a person may land in gaming disorder [31]. This helps us understand that an optimum level of engagement is a significant consideration while designing serious games for mental health promotion and management of mental health disorders [32].

Persuasive games: a growing trend in mental health promotion

Persuasive games differ from traditional serious games in that they motivate people to change their attitudes, beliefs, and behavior through gamified video game elements [33]. This has led to many game developers developing video games to create awareness of various mental health problems focused on compassion and empathy. This is achieved by providing an experience that makes the players look deep into the heart and soul of the character they are playing inside the video game or the virtual world [34]. For example, the video game “Homeless: It’s No Game” was developed to motivate people to sympathize with the elderly and homeless. A study done by Lavender found mixed results, but some people who played video games developed an increased level of empathy [35]. A study done by Khaled et al. developed and tested a persuasive video game, “Smoke?” to persuade people to stop smoking and create awareness of health problems related to tobacco consumption. One of the major issues in designing persuasive games was not introducing too many concepts and information and overloading the players. Also, the player must be able to replay video game segments to understand different outcomes based on their actions [36]. More research is needed to understand and effectively utilize this type of gamification in the management of mental health problems like workplace stress, anxiety, and deaddiction for substance use disorders.

## Conclusions

Gamification, serious games, and VR interventions in mental health face a significant challenge in acceptance, adherence, and having the necessary skill set to engage in that particular activity. The lack of scientific ground for various serious games also poses a significant challenge, as a problem arises if they are being developed in collaboration between psychologists, psychiatrists, and game developers. Since mental health problems are stigmatized worldwide, measures could be taken in the form of extensive surveys in different cultural and social strata to understand the needs of different demographics, their ability to handle gamified interventions, their social and cultural beliefs, and the need to tailor the serious games to their needs grounded with scientific evidence.

From the planning stage of a serious video game, the various mechanics, like color theory for visual calmness, music designed for serenity and relaxation, easy-to-master controls, and game mechanics designed in collaboration with experts in the field of mental health, tailored toward the needs of the particular population, rigorously tested by trials, will help in the development of a non-judgmental and unbiased video game application that could help in inducing behavior change among people suffering from mental health problems and will also improve adherence to mental health-seeking behavior. Future research studies emphasizing the above points could help develop gamified video game interventions for managing mental health problems grounded on scientific bases and catering to the social and cultural beliefs of the target population.
